# Disordered speech disrupts conversational entrainment: a study of acoustic-prosodic entrainment and communicative success in populations with communication challenges

**DOI:** 10.3389/fpsyg.2015.01187

**Published:** 2015-08-12

**Authors:** Stephanie A. Borrie, Nichola Lubold, Heather Pon-Barry

**Affiliations:** ^1^Human Interaction Lab, Department of Communicative Disorders and Deaf Education, Utah State University, Logan, UT, USA; ^2^School of Computing, Informatics, and Decision Systems Engineering, Arizona State University, Tempe, AZ, USA; ^3^Department of Computer Science, Mount Holyoke College, South Hadley, MA, USA

**Keywords:** conversational entrainment, spoken dialogue, human interaction, speech pathology, disordered speech, accented speech, communication success

## Abstract

Conversational entrainment, a pervasive communication phenomenon in which dialogue partners adapt their behaviors to align more closely with one another, is considered essential for successful spoken interaction. While well-established in other disciplines, this phenomenon has received limited attention in the field of speech pathology and the study of communication breakdowns in clinical populations. The current study examined acoustic-prosodic entrainment, as well as a measure of communicative success, in three distinctly different dialogue groups: (i) healthy native vs. healthy native speakers (Control), (ii) healthy native vs. foreign-accented speakers (Accented), and (iii) healthy native vs. dysarthric speakers (Disordered). Dialogue group comparisons revealed significant differences in how the groups entrain on particular acoustic–prosodic features, including pitch, intensity, and jitter. Most notably, the Disordered dialogues were characterized by significantly less acoustic-prosodic entrainment than the Control dialogues. Further, a positive relationship between entrainment indices and communicative success was identified. These results suggest that the study of conversational entrainment in speech pathology will have essential implications for both scientific theory and clinical application in this domain.

## Introduction

The ability to converse with others is one of the most important communication skills in daily living. Through spoken interaction, we are able to transmit and receive knowledge as well as establish and maintain interpersonal relations. Clinical communication disorders characterized by deficits in speech production and/or speech perception, including dysarthria, apraxia of speech, stuttering, vocal pathologies, hearing impairment, and even other communication challenges such as foreign-accents, frequently result in conversational breakdowns. Such breakdowns are known to negatively impact quality of life in many essential domains including vocation, social functioning, and mental health status (e.g., Hustad et al., 1998; Craig et al., 2009).

Across decades and disciplines, there is a large and growing body of evidence demonstrating that *conversational entrainment*, the propensity for dialogue partners to modify their behavior to more closely align with one another, is critical for successful interaction. Operationally defined as the “spatiotemporal coordination resulting from rhythmic responsiveness to a perceived rhythmic signal” (Phillips-Silver et al., 2010, p. 5), this communication phenomenon is observed at multiple levels of linguistic representation (e.g., Brennan and Clark, 1996; Branigan et al., 2000; Pardo, 2006; Lubold and Pon-Barry, 2014), as well as in non-verbal behaviors such as gesture, body posture, and eye gaze (e.g., Shockley et al., 2003; Louwerse et al., 2012). Entrainment has been shown to support communication success, facilitating sense making and the exchange of information (Garrod and Pickering, 2004; Gill, 2012), as well as establishing affiliation, rapport, and intimacy during social interaction (e.g., Chartrand and Bargh, 1999; Lee et al., 2010). For example, Nenkova et al. (2008) reported that lexical entrainment on high frequency words was correlated with task success in a series of computer games that required dialogue partners to work together, using verbal communication, to solve. Thus, conversational entrainment appears to function as a “…powerful coordinating device…to optimize comprehension, establish social presence, and create positive and satisfying relationships” (Borrie and Liss, 2014, p. 816). Accordingly, deficits in entrainment are likely to be problematic for conversational success and quality of life.

Despite the importance of conversational entrainment to successful human interaction, the phenomenon of aligning verbal behavior has received limited attention in the field of speech pathology and the study of communication disorders. Borrie and Liss (2014), however, recently proposed that because of the pathological rhythms, entrainment deficits are likely a common feature of a range of communication disorders. The authors speculate that such deficits “may reveal an important, yet unexplored, source of communication difficulty and demise in quality of life” in these clinical populations.

As a first step to exploring conversational entrainment with pathological speech disorders, Borrie and Liss (2014) examined whether the presence of neurologically degraded speech, dysarthria, had any measureable effect on the spoken productions of healthy subjects. This study employed a quasi-conversational experimental turn-taking paradigm in which healthy individuals were required to listen and respond to audio recordings of disordered speech characterized by abnormal rhythmic production parameters. The authors observed that the healthy subjects subconsciously modified their speech rate and pitch variation in the direction of the pathological stimuli, supporting examination of conversational entrainment as a viable target of investigation in the domain of speech pathology. The next step in this line of work is to examine conversational entrainment in embodied face-to-face interaction, allowing this communication phenomenon to be explored as a property of the dialogue pair—a dynamic joint activity in which both parties must coordinate elements of their verbal productions to succeed (Clark, 1996).

The current study investigates the empirical basis for the hypothesis that unfamiliar or pathological rhythmic production parameters disrupt conversational entrainment in embodied face-to-face interaction, and further, that these disruptions interfere with the success of the interaction. We use foreign-accented speech and the neurological speech disorder of dysarthria as test cases for entrainment in interactions involving unfamiliar and pathological speech production parameters, respectively. Foreign-accented speech introduces unfamiliar but regular pronunciation and rhythmic patterns as the English is overlaid on the native language structure. Dysarthria, on the other hand, is the result of damage to the motor speech system, which physically impairs movement of the muscles used to produce speech. The speech deficits in dysarthria manifest as more varied and unpredictable, “…phonemes produced adequately in one context may be distorted or omitted in the next word, speech may deteriorate in a mumbled rush of speech at the end of a sentence, and voicing may break or cease intermittently” (Borrie et al., 2012, p. 2). Given the more variable nature of pathological speech, it is hypothesized that entrainment in interactions involving dysarthric speakers will be more impaired than entrainment in interactions involving accented speakers.

Specifically, we examine acoustic-prosodic entrainment in spoken interactions involving healthy native speakers interacting with individuals with either Chinese-accented or dysarthric speech; we include interactions involving two healthy native speakers of American English for comparative purposes. If the entrainment that characterizes dialogues involving accented and/or disordered speakers is less notable than that in dialogues involving healthy speakers, then there is support for the idea that accented and/or degraded speech induces some level of entrainment deficit into typical human interaction. Further, if entrainment deficits correlate with a measure of communicative success, then we have evidence to suggest that acoustic-prosodic entrainment is indeed an essential element to address in management of such deficits. Such finding would support large-scale studies of conversational entrainment in clinical populations and, together, would lay the foundation for the development of a novel diagnostic tool to detect, quantify, and characterize entrainment deficits—as a source of conversational breakdown—in clinical populations, and, ultimately, identify potential treatment targets for remediation of deficits.

## Materials and Methods

### Study Overview

Sixty dyads—differentiated into one of three dialogue groups by the nature of the individuals involved—engaged in an interaction task in which they were required to collaborate verbally to identify differences between pairs of pictures. Measures of four acoustic-prosodic features including average pitch, intensity, jitter, and shimmer (Jurafsky and Martin, 2008) were extracted for each speaking turn for each individual during the course of each interaction. A multi-level entrainment analysis was used to examine measures of synchrony and proximity (defined below) for each dialogue groups. A measure of communicative success was also calculated for each of the dialogues. This study was carried out with ethical approval from the Institutional Review Board at Utah State University.

### Participants

The speech corpus analyzed here was collected from 120 participants comprising 80 healthy individuals, 20 individuals with disordered speech, and 20 individuals with accented speech. The healthy individuals were all native speakers of American English with no neurological history or presence of pathological speech patterns. The individuals classified as *disordered* were also native speakers of American English but had a clinical diagnosis of a mild dysarthria (of various etiologies), as evaluated by a Speech-Language Pathologist, experienced in the assessment and differential diagnosis of motor speech disorders. Mild was defined as a score between 80 and 95% words correct on the Sentence Intelligibility Test (SIT; Yorkston et al., 1996). Individuals with dysarthria were excluded from the study if concomitant impairments in speech (i.e., apraxia of speech) or language (i.e., aphasia) were identified. Individuals classified as *accented* were born and raised (till at least 15 years of age) in China and spoke English with a strong Chinese-accent. All 120 participants were aged between 20 and 50 years (*M* = 29.64, SD = 3.21) and reported no significant history of language, hearing, or cognitive disabilities. In addition, the healthy native individuals reported no significant contact with persons having dysarthria or persons born and raised in China.

Participants were divided into sixty dyads to form three distinct dialogue groups (*n* = 20), with dyads consisting of one of the following combinations: (i) two healthy native individuals (Control), (ii) one healthy native individual and one individual with a Chinese-accent (Accented), and (iii) one healthy native individual and one individual with dysarthria (Disordered). Dyads were matched for gender and age (group means within 1 SD of each other), as closely as possible, across the three dialogue groups.

### Procedure

Each dyad participated in a single experimental recording session. The experimental recording sessions were held in either the Human Interaction Lab at Utah State University or the Motor Speech Disorders Lab at Arizona State University. Upon entering the lab, participants were seated at opposite ends of a table (facing one another) and fitted with a wireless CVL Lavalier microphone, synced with a Shure BLX188 DUAL Lavalier System connected to a Zoom H4N Portable Digital Recorder. Separate audio channels and standard settings (48 kHz; 16 bit sampling rate) were employed.

Stimuli and instructions for the interaction task, the Diapix Task (Van Engen et al., 2010), were then administered. Each participant was given one of a pair of pictures and was instructed to hold their picture at an angle at which it would not be visible to the person sitting across the table from them. The pair of pictures depicted virtually identical scenes (i.e., farm yard, beach trip), differing from one another by 10 small details (i.e., color of boots, number of waves). The dyad was then told that their goal was to work together, simply by speaking to one another, to identify the 10 differences between the pair of pictures. They were instructed to complete the task as quickly and accurately as possible. No additional rules (i.e., who could talk when) or roles (i.e., giver, receiver) were given—dyads were free to verbally interact in any way they saw fit in order to achieve the instructed goal. Dyads did this task up to three times (using a different pair of pictures each time), only progressing to the next picture set when all 10 differences had been identified, to enable the collection of at least 12 min of semi-spontaneous spoken interaction. Following this, the audio recording equipment was turned off, and participants were debriefed and thanked before leaving the lab. The audio recording of the interaction was then transferred to a computer and edited down to exactly 12 min of spoken dialogue.

### Data Preparation

The total data set consisted of 60 audio recordings, consisting of 12-min interaction sound (.wav) files. These 60 interaction files included 20 interactions in the Control group, 20 interactions in the Disordered group, and 20 interactions in the Accented group. Using the interaction files, three trained research assistants used acoustic analysis software, Praat (Boersma and Weenink, 2014), to manually annotate each interaction for individual speaking turns. The beginning of each speaking turn is identified as the moment that a participant begins articulating an utterance and ends when articulation ceases. Simultaneous illustrations of the associated spectrograms were used to aid annotation accuracy—especially important when labeling pathological speech. Overlapping speech results in overlapping turns.

### Data Analysis

#### Entrainment Analysis

Utilizing the segmented .wav files, four acoustic features were computed for each speaking turn: average intensity, average pitch, average jitter, and average shimmer. These features were computed using OpenSmile (Eyben et al., 2010). The pitch for male speakers was normalized by scaling the average pitch to lie in the same range as the female values; all other acoustic-prosodic features were left raw. Using this data, we computed two types of acoustic-prosodic entrainment scores—*synchrony* and *proximity*. Our approach considers entrainment to be a local phenomenon occurring on a turn-by-turn basis and provides a conversation-level score that reflects the amount of entrainment for a single acoustic feature throughout the conversation. The measures of synchrony^1^ and proximity are summarized below and are described in more detail in Lubold and Pon-Barry (2014).

Two speakers exhibit synchrony when they modulate their acoustic features in tandem (see also Levitan and Hirschberg, 2011). For example, two speakers may have very different raw feature values for their average intensities, but as they converse, each speaker’s individual changes in intensity move in parallel. When one speaker increases how loud they are speaking, the other speaker is also increasing their loudness. A synchrony score is generated for each dyad by measuring the Pearson’s correlation coefficient between raw feature values of the two speakers at adjacent turns and then testing for significance with a two-tailed *t*-test. To facilitate interpretation, the resulting correlation coefficient is linearly scaled to fall in the range [0,1]. Higher scores represent a higher degree of entrainment.

Proximity is a form of entrainment based on how similar two speakers are to one another at each turn in contrast with the rest of the interaction. Consider the case of pitch proximity. For every conversation turn, we compute Δ*adjacent*, the absolute difference between Speaker A’s pitch in the utterance and Speaker B’s pitch in the next utterance. We then compute Δ*other*, an average of absolute differences between Speaker A’s pitch in the utterance and Speaker B’s pitch in utterances from 10 non-adjacent turns. We compute these values for each turn in the conversation, then run a paired-samples *t*-test between the Δ*adjacent* and Δ*other* values. The proximity score for the whole conversation is taken as the absolute value of the resulting *t*-value. Proximity scores therefore have a lower bound of 0 and no upper bound; higher proximity scores indicate a higher degree of entrainment.

$$\begin{array}{ccc} \Delta adjacent & = & \left|Turn_{a\left(i\right)}-Turn_{b\left(i\right)}\right|\\ \Delta other & = & \frac{\sum_{j=1,j\neq i}^{10}\left|Turn_{a\left(i\right)}-Turn_{b\left(i\right)}\right|}{10} \end{array}$$

Synchrony and proximity serve as the two entrainment scores utilized in the following evaluations. A single synchrony score captures the turn-by-turn synchronous changes of a low-level acoustic feature within a conversation dyad. A single proximity score captures the turn-by-turn proximal changes of a low-level acoustic feature within a conversation dyad. Four acoustic-prosodic features were examined: average intensity, average pitch, average jitter, and average shimmer. This results in eight entrainment index scores for each dyad, four for synchrony and four for proximity, which form the basis of the subsequent evaluations.

#### Analysis of Communicative Success

A measure of communicative success for each dialogue was computed in the following way. As outlined in the procedure, the interaction task required dialogue partners to work together, as quickly and accurately as possible, to identify the differences between pairs of pictures. Each pair of pictures contained 10 differences and, as soon as all 10 differences were identified, dyads were given another pair of pictures to work on. This occurred up to three times. Some dyads however did not make it past the first picture pair. Total number of differences identified in 12 min of spoken interaction was then used as a simple, gross measure of communicative success: relatively low and high numbers of identified differences indicate relatively high and low communicative success, respectively. Thus, communicative success is essentially an evaluation of task success, accounting for how efficiently the dyad used verbal communication to collaboratively work through the given task.

## Results

### Entrainment Index Scores

Descriptive statistics describing the acoustic-prosodic entrainment, in terms of synchrony and proximity entrainment scores, for each dialogue group, are reported in Table 1. These average entrainment index scores suggest that the dialogue groups did not entrain to the same degree. With both synchrony and proximity, we see a clear trend demonstrating that the Control group entrained the most, the Disordered group entrained the least, and the Accented group, somewhere in between.

**TABLE 1:** **Descriptive statistics for each entrainment index score by group**.

**Entrainment type**	**Feature**	**Disordered**		**Accented**		**Control**	
		***M***	**SD**	***M***	**SD**	***M***	**SD**
Synchrony	Intensity	0.53	0.06	0.57	0.05	0.59	0.06
	Pitch	0.53	0.03	0.54	0.03	0.57	0.05
	Jitter	0.51	0.04	0.51	0.04	0.54	0.05
	Shimmer	0.51	0.05	0.52	0.04	0.52	0.04
Proximity	Intensity	1.56	1.79	1.81	1.18	1.91	1.53
	Pitch	0.76	0.92	1.18	0.94	1.58	0.90
	Jitter	0.81	0.82	1.30	0.68	1.37	1.16
	Shimmer	1.10	0.81	1.49	0.80	1.13	0.67

A series of one-factor analysis of variance (ANOVA) calculations were used to examine the significance of group differences in entrainment index scores and results are reported in Table 2. For synchrony, the analysis revealed statistically significant group differences in the scores for intensity, pitch, and jitter. The eta-squared (η^2^) effect size indicated that group membership explained 14% of the variance in synchrony for intensity, 13% for pitch, and 11% for jitter. For intensity, this is a borderline large effect size by conventional standards; for pitch and jitter, these are medium effect sizes (Tabachnick and Fidell, 2001). *Post hoc* tests (Tukey’s honest significant difference, HSD; *p* < 0.05) indicated that the Disordered group entrained significantly less than the Control group on intensity, *t*(38) = 0.12, *p* < 0.001, pitch, *t*(38) = 0.07, *p* < 0.005, and jitter, *t*(38) = 0.07, *p* < 0.005. The significant differences between the Disordered and the Control group were commensurate with large effect sizes, with Hedges *g* > 0.80. In all of these entrainment indices, the Accented group was not significantly different from either the Control or the Disordered group.

**TABLE 2:** **ANOVA results for between groups comparison of entrainment index scores**.

**Entrainment type**	**Feature**	***F***	**Effect size (η^2^)**	***P***
Synchrony	Intensity	5.49**	0.14	0.007
	Pitch	4.67*	0.13	0.013
	Jitter	3.73*	0.11	0.030
	Shimmer	0.35	0.01	0.707
Proximity	Intensity	1.63	0.06	0.205
	Pitch	3.69*	0.12	0.031
	Jitter	1.77	0.06	0.180
	Shimmer	2.81	0.09	0.069

*p < 0.05. **p < 0.01.

For proximity, the analysis revealed statistically significant group differences for the pitch scores. The η^2^ effect size indicated that group membership explained 12% of the variance in proximal-type entrainment for pitch, which is a medium effect size by conventional standards (Tabachnick and Fidell, 2001). *Post hoc* tests (Tukey’s HSD) revealed that the Disordered group entrained significantly less than the Control group on pitch, with a *t*-value significantly closer to 0, *t*(38) = 0.09, *p* < 0.05. The significant difference between the Disordered and the Control group was commensurate with a large effect size, with Hedges *g* > 0.80. In all of these entrainment indices, the Accented group was not significantly different from either the Control or the Disordered group.

### Entrainment and Communicative Success

Descriptive statistics of communicative success for each dialogue group are reported in Table 3. These scores revealed that the Disordered group had the lowest average communicative success scores, followed by the Accented group. The Control group achieved the highest average communicative success score. An ANOVA demonstrated that these differences were significantly different, *F*(2,57) = 232.05, *p* < 0.001, and *post hoc* tests (Tukey’s HSD) indicated that all three groups were significantly different from each other on this measure.

**TABLE 3:** **Descriptive statistics for communicative success by group**.

****	**Disordered**		**Accented**		**Control**	
	***M***	**SD**	***M***	**SD**	***M***	**SD**
Communicative success	9.11	0.81	13.25	1.33	17.85	1.53

**FIGURE 1:**
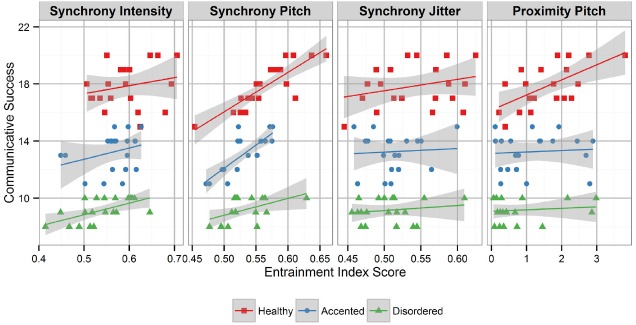
**Acoustic-prosodic entrainment and communicative success: from left to right, the panels reflect significant correlations between communicative success and entrainment index scores of synchrony intensity, synchrony pitch, synchrony jitter, and proximity pitch**.

Given the nature of the dyads—a continuum of speech deviating from typical norms—Pearson’s correlation coefficients with two-tailed *t*-tests were used to examine the relationship between the entrainment index scores and the measure of communicative success across the dialogue groups (Thomason et al., 2013). Results are reported in Table 4 and illustrated in Figure 1. In brief, the correlation analysis revealed a significant relationship between communicative success and synchrony entrainment scores for pitch, intensity, and jitter and a significant relationship between communicative success and the proximity entrainment scores for pitch. Thus, the entrainment indices that correlate with communicative success are the same dimensions that differentiate Disordered dialogues from Control dialogues.

**TABLE 4:** **Communicative success and entrainment across groups**.

**Feature**	**Synchrony**		**Proximity**	
	***r***	***P***	***r***	***P***
Intensity	0.43**	<0.001	–0.95	0.476
Pitch	0.57**	<0.001	–0.54**	<0.001
Jitter	0.36*	0.005	–0.23	0.087
Shimmer	0.11	0.392	–0.20	0.649

*p < 0.005. **p < 0.001.

The relationship between the entrainment scores and the measures of communicative success within each dialogue group were examined with Pearson’s correlation coefficients with two-tailed *t*-tests. Results of the within group analysis revealed a significant relationship between the synchrony entrainment scores of pitch and the communicative success measures for the Disordered, *r*(18) = 0.53, *p* = 0.02, Accented, *r*(18) = 0.81, *p* < 0.001, and Control, *r*(18) = 0.85, *p* < 0.001, groups. Correlations with other entrainment indices were not significant in the within group analysis.

## Discussion

Conversational entrainment is a well-established phenomenon in spoken interactions involving typical speakers. The current study examined acoustic-prosodic entrainment in spoken interactions involving typical speakers and individuals with unfamiliar or pathological production parameters, including Chinese-accented and dysarthric speech. Dialogue group comparisons revealed that the level of entrainment, as measured in terms of synchrony and proximity, was differentiated by the nature of the dyad. Recall that synchrony is present when dialogue partners modulate their acoustic-prosodic parameters in parallel. The results showed that the Disordered dialogues were characterized by significantly less synchrony than the Control dialogues on features of average intensity, average pitch, and average jitter. These findings, therefore, demonstrate that dialogues involving people with neurological speech disorders are less “in sync” than dialogues involving two healthy native speakers of American English. The second measure of entrainment, proximity, captures similarity of the dialogue partners’ acoustic-prosodic features at each turn boundary. With this analysis, the results found that the Disordered dialogues were characterized by significantly less proximity than the Control dialogues on the feature of average pitch. This indicates that, on a local, turn-by-turn basis, the dyads in the Control group are aligning their pitch more closely than the dyads in the Disordered group. While group differences in proximity of average intensity, average jitter, and average shimmer were not significant, clear trends of less proximity in the Disordered group, relative to the Control group, were evident with all of these features (see Table 1). It does, however, appear that along proximal dimensions of entrainment, mean pitch may be the most informative acoustic-prodosic feature in differentiating dialogues involving individuals with disordered speech from dialogues that transpire with typical healthy populations.

Group differences in entrainment indices of the Accented dialogues were not significant, however proximity and synchrony scores for this group trended toward an intermediary position between the Disordered and Control dialogues. This pattern corroborates our prediction that while the unfamiliar production parameters that characterize accented speech may disrupt entrainment to some degree, entrainment in interactions involving dysarthric speakers, and the more variable production patterns associated with pathological speech, would be more impaired. This suggests that acoustic-prosodic entrainment may become increasingly difficult to achieve as the production parameters of one’s dialogue partner deviate further and more variably from the typical native norms—a speculation which calls for further exploration of the variables that influence the tendency to entrain. Certainly, future studies that include individuals with more severely impaired speech, including moderate and severe dysarthria, would advance our understanding of the underlying mechanisms.

Taken together, the results of the synchrony and proximity analysis indicate that, on particular features, the Disordered dialogues are indeed characterized by significantly less acoustic-prosodic entrainment than the Control dialogues, suggesting that the presence of pathological speech production parameters induces some level of entrainment deficit in embodied face-to-face spoken interaction. One theoretical model to explain the observed findings is the sensorimotor theory of “beat induction,” proposed by Todd et al. (2002) and expanded to the social domain by Phillips-Silver et al. (2010). According to this model, entrainment is comprised of three critical components: rhythmic action/production, rhythmic detection/perception, and rhythmic integration (i.e., adjusting productions based on perceptions). Consequently, a breakdown in any or all components of this model will disrupt entrainment. Given the prevalence of rhythmic deficits across a number of communication disorders including that of dysarthria, Borrie and Liss (2014) recently speculated that disruptions in entrainment are likely to be present in clinical populations. The current study is the first of its kind to yield confirmatory evidence that the presence of the neurological speech disorder, dysarthria, does indeed disrupt acoustic-prosodic entrainment in embodied face-to-face spoken interaction. The next important step in this line of work is to empirically examine the locus of entrainment deficits in spoken dialogue, parsing out contributions on behalf of the individual with dysarthria and that of the healthy dialogue partner.

This study also found that entrainment is strongly correlated with interaction success. Across all dialogues, the results showed that the degree of entrainment, on particular indices, was significantly correlated with a single gross measure of communicative success—accounting for how efficiently information was transferred between the dyad. Specifically, a positive relationship with communicative success was evident with all four of the indices in which we also observed significant group differences (intensity synchrony, pitch synchrony, jitter synchrony, and pitch proximity). That is, the more entrained a dyad was on each of these dimensions, the more successful the dyad was at using speech to collaboratively work through the demands of the interaction task. That entrainment correlates with a measure reflective of communication success with healthy populations has been observed prior (e.g., Reitter and Moore, 2007; Friedberg et al., 2012) however, to our knowledge, this is the first study of its kind to explicate this type of relationship with a pathological communication disorder. Literature in cognitive psychology suggests that linguistic alignment may be an external manifestation of a jointly built and understood situation model, a conceptual representation of relevant aspects of the situation under discussion (Garrod and Pickering, 2004), and that mental model sharing can significantly improve team effectiveness (e.g., Mathieu et al., 2000). Accordingly, entrainment deficits may reflect a breakdown in converging on a common situation model, and result in conversational breakdowns (Branigan et al., 2011).

This relationship between entrainment and interaction success was also examined within each of the three dialogue groups, accounting for overall group difference in the measure of communicative success. With this analysis, we see that pitch synchrony is the critical entrainment dimension in predicting success in the interactions—a finding that is robust in all three of the dialogue groups. While more research is needed to elucidate this relationship, this finding sheds light on a completely novel but potentially valuable metric in the management of neurological speech disorders. That is, the quantification of pitch synchrony between dialogue partners may allow clinicians to generate predictions regarding the level of success, or lack thereof, in spoken interactions with specific communication partners (e.g., speaker with dysarthria and their spouse). Furthermore, treatments that focus on improving pitch synchrony within a specific dyad may prove key to improving interaction success with people with neurological speech disorders.

To continue to build upon this line of work, a number of important steps are required. In addition to those directions already raised, we must expand the acoustic-prosodic analysis to include a more comprehensive set of speech rhythm metrics (e.g., Liss et al., 2009). It is also crucially important to address how entrainment deficits relate to other measures of communicative success, particularly those that pertain to social and emotional functions including the development and maintenance of interpersonal relations. For example, Lee et al. (2010) showed that the degree to which married couples entrain on prosodic dimensions during a problem-solving task is correlated to perceptual judgments of more positive and fulfilling interactions. Further, whether the entrainment deficits observed in a problem-solving task are the same as those that may be evident in a more naturalist conversation warrants exploration. Building an empirically-based model of conversational entrainment in disordered settings has significant clinical implications. The current findings, in combination with future studies, could inform the development of a novel diagnostic tool to reveal and understand a currently undiagnosed source of conversational breakdown in pathological speech disorders. Further, this objective tool could be used to identify potential treatment targets for remediation of deficits (i.e., training on entrainment dimensions that correlate most strongly with communicative success). Large scale clinical trails on whether improving entrainment on important acoustic-prosodic features could improve spoken interaction, would then ensue. Taken together, such studies could make a significant contribution, and/or constitute an augmentation strategy, to the existing behavioral therapies dedicated to improving communication in people with dysarthria. Thus, the significance of continuing to investigate entrainment with this clinical population cannot be over estimated.

Finally, the study of acoustic-prosodic entrainment in clinical populations should not be limited to populations with dysarthria. As per the previously mentioned theoretical framework of entrainment (Todd et al., 2002; Phillips-Silver et al., 2010), impairments in entrainment arise when there are deficits in the capacity to produce, perceive, and/or integrate rhythmic information. Thus, entrainment investigations in communication disorders characterized by rhythmic deficits such as apraxia of speech, stuttering, voice disorders, and hearing impairment, to name just a few, are well justified. Employing a more multi-level analysis that includes entrainment at other levels of linguistic representation—lexical, syntactic, semantic—as well as including other important conveyers of communicative behavior such as body movement, facial expression, and eye gaze are also important future directions.

## Conclusion

In sum, the current study demonstrates that entrainment indices were less pronounced in interactions involving people with dysarthria, as compared to interactions involving two healthy native speakers of American English, suggesting that the pathological acoustic-prosodic production parameters that characterize neurological speech disorders may disrupt entrainment—the rhythmic coordination phenomenon that connects two people in time and space during human interaction. Further, there is evidence that these entrainment disruptions, particularly pitch synchrony, may negatively impact dialogue success. These findings support the prediction that the presence of clinical communication disorders may cause entrainment impairments and contribute to the conversational breakdowns evidenced in these populations. Research going forward will explicate this relationship further, with the ultimate goal of developing and testing quantitative tools to address entrainment impairments, clinically.

### Conflict of Interest Statement

The authors declare that the research was conducted in the absence of any commercial or financial relationships that could be construed as a potential conflict of interest.

## Footnotes

1 We use the term synchrony to describe changes in raw acoustic features of two speakers that are “in sync” at the dialogue turn-level. This should not be interpreted to mean that the two speakers are speaking at the exact same moment.
